# Camera trap placement for evaluating species richness, abundance, and activity

**DOI:** 10.1038/s41598-021-02459-w

**Published:** 2021-11-29

**Authors:** Kamakshi S. Tanwar, Ayan Sadhu, Yadvendradev V. Jhala

**Affiliations:** Wildlife Institute of India, Chandrabani, Dehradun, 248001 India

**Keywords:** Ecology, Conservation biology

## Abstract

Information from camera traps is used for inferences on species presence, richness, abundance, demography, and activity. Camera trap placement design is likely to influence these parameter estimates. Herein we simultaneously generate and compare estimates obtained from camera traps (a) placed to optimize large carnivore captures and (b) random placement, to infer accuracy and biases for parameter estimates. Both setups recorded 25 species when same number of trail and random cameras (n = 31) were compared. However, species accumulation rate was faster with trail cameras. Relative abundance indices (RAI) from random cameras surrogated abundance estimated from capture-mark-recapture and distance sampling, while RAI were biased higher for carnivores from trail cameras. Group size of wild-ungulates obtained from both camera setups were comparable. Random cameras detected nocturnal activities of wild ungulates in contrast to mostly diurnal activities observed from trail cameras. Our results show that trail and random camera setup give similar estimates of species richness and group size, but differ for estimates of relative abundance and activity patterns. Therefore, inferences made from each of these camera trap designs on the above parameters need to be viewed within this context.

## Introduction

Reliable estimation of species richness, abundance, activity and subsequent monitoring play a pivotal role in achieving specific conservation goals through evidence-based management^1^. However, selection of suitable techniques requires *a-priori* assessment of their accuracy, precision, replicability, and cost-effectiveness to meet the desired objectives before the technique is recommended on a large scale. Camera traps have been widely used as a wildlife monitoring tool due to their objectivity, ease of use, and ability to generate information on a large spectrum of species^2^. Camera trapping surveys are primarily designed to document species richness^3^, occupancy^4^, abundance indices^5,6^, estimate abundance of individually identifiable species in capture-recapture framework^7–10^ and determine their activity patterns^11^. However, with the technological advances, researchers started using camera traps to study population ecology^12^, camera trap-based distance sampling^13^, behavior^14^, forest ecology^15^ and carrying out conservation assessments^16^.

A basic assumption of all inferences from camera trap studies is that the data generated are unbiased representation of underlying parameters (of species richness, abundance, temporal activity, either after correcting for effort^7^ and/or detection^9,17^). However, a typical capture-recapture study is designed to maximize detections of the target species and is essentially non-random and often not systematic^18^. Such camera trap designs also generate secondary data on several non-target species which are often used to infer their relative abundance indices^19–21^, activity patterns^22,23^, and occupancy estimates^24^. However, these inferences on the non-target species can be biased due to the sampling design and camera placement. Due to differential use of trails by different species^25^, biases can occur in estimating relative abundance, group size, and temporal activity^21^. In a review of 266 camera trap studies, Burton et al.^18^ found 47.6% of studies using the same surveys to estimate variables of non-target species e.g. occupancy, relative abundance, and activity pattern. Attempts to evaluate if such designs result in a biased inference and of what magnitude have been few^26–30^. Di Bittetti et al.^26^ and Blake and Mosquera^27^ have summarized that a combination of trail and off-trail cameras will provide a comprehensive picture of species composition and their relative abundance. While citing the above-mentioned approach Cusack et al.^28^, Wearn et al.^31^ and Kolowski et al.^29^ described difficulties in selecting the proportion and spatial distribution of these trail and random locations in a systematic sampling design. Thus, they recommended the use of random camera setup and emphasized its importance in sampling microhabitats. However, they also showed that in order to eliminate biases in inferences made at the community level and overcome lower capture rates from random design, a large sampling effort would be required.

Herein, we deployed camera traps on trails to maximize photo-captures of tigers (*Panthera tigris*) and leopards (*Panthera pardus*) (trail cameras), and sampled simultaneously the same extent with randomly placed camera traps (random cameras). We computed the rate of species accumulation, species richness, relative abundance, detection probability, group size and daily activity pattern of large and medium sized terrestrial mammals using both camera placements and compared their outcomes. We also calculated density estimates of ungulates from distance sampling and of tiger and leopards from spatially explicit capture-mark-recapture and regressed them against RAI values obtained from trail and random cameras. This experimental setup permits us to test if camera trap placement is an important aspect to be considered for estimating species richness, abundance, and activity.

## Methods

### Study area

The study was carried out in Ranthambhore National Park, (76.23 E to 76.39 E and 25.84 N to 26.12 N) situated in the semi-arid part of western India. The terrain is rugged and hilly, interspersed with valleys and plateaus which makes for largely two types of habitat i.e. woodland and savannahs. The area is dominated with tropical dry deciduous forest (dominated with *Anogeissus pendula*) and scrubland-thorn forests (dominated with *Grewia flavescens, Capparis sepiaria*). Ranthambhore experiences sub-tropical dry climate with hot and dry summer (March–June), moderately wet monsoon (July–September) and dry winter (October–February). A small solitary stream along with man-made lakes and water holes manages to sustain the faunal assemblage of the park through the dry months. The flagship species of Ranthambhore National Park is the tiger and it serves as the source population of tiger in the semi-arid landscape of western India^32^. Other large carnivores include leopard, striped hyena (*Hyaena hyaena),* and sloth bear (*Melursus ursinus*). Meso-carnivore guild comprised of jungle cat (*Felis chaus*), golden jackal (*Canis aureus*), caracal (*Caracal caracal*), desert cat (*Felis silvestris*), rusty spotted cat (*Prionailurus rubiginosus*), fox (*Vulpes bengalensis*), and honey badger (*Mellivora capensis*). Small carnivores include small Indian civet (*Viverricula indica*), Asian palm civet (*Paradoxurus hermaphroditus*), ruddy mongoose (*Herpestes smithii*), Indian grey mongoose (*Herpestes edwardsii*), and small Indian mongoose (*Herpestes auropunctatus*). Herbivores includes spotted deer (*Axis axis*), sambar (*Rusa unicolor),* blue bull (*Boselaphus tragocamelus*), Indian gazelle (*Gazella bennettii*), wild pig (*Sus scrofa*), gray langur (*Presbytis entellus*), rhesus macaque (*Macaca mulatta*), black-naped hare (*Lepus nigricollis*), Indian crested porcupine (*Hystrix indica*), and peafowl (*Pavo cristatus*). For species richness we also included squirrels, monitor lizards and birds (grouped into two: ground dwelling and other birds) in our analysis. The National Park area of Ranthambhore is mostly inviolate, however, in the peripheral areas herders often breach the boundary wall and push their cattle inside the Park for grazing. We therefore also recorded all domestic and feral livestock (cattle, buffalo, goats, camels, donkeys, and dogs) that were photo-captured.

### Field method

#### Camera trapping

The study area was divided into grids of 2 km^2^ for systematic deployment of camera traps for both the placements. Trail cameras were deployed targeting population estimation of tigers and leopards in a mark-recapture framework and were positioned at locations to maximize their photo-captures. Tigers and leopards mostly use forest roads, animal trails, dry river beds, and fire lines to patrol their territories and to commute^33^. After a reconnaissance survey for carnivore signs and usage, a pair of camera traps was deployed at the most suitable locations within each grid to photo-capture tigers and leopards (October to December 2018). Trail cameras (Cuddeback™, WI5411 USA) were deployed at 106 locations, and operated for 25 days constituting an effort of 3537 trap day (no. of cameras × no. of operational days). Cameras were tied to a pole/tree at the height of 30–45 cm from the ground, and placed 3–5 m away from the middle of the trail to ensure full-body capture of the target animals. The time delay between successive pictures was kept as ‘Fast as Possible’ mode (1–2 s delay), however, at night the delay increased to 8–10 s depending on the battery conditions (which is required to recharge the white light flash).

For the random design, 31 infrared flash cameras (Reconyx^®^ Hyperfire HC500, WI 54636USA) were placed at random locations with (centroids of the sampling grids) a fixed bearing to maintain a random field of view. The camera height was kept at 30–45 cm above ground. The ‘No delay’ setting of the camera allowed it to take consecutive pictures without any lag. Random cameras were operated for 40 days constituting an effort of 1035 trap days, each camera was visited after 5–7 days to check their set up, battery status and to download the data.

### Analytical methods

#### Species richness and accumulation

**Figure 1:**
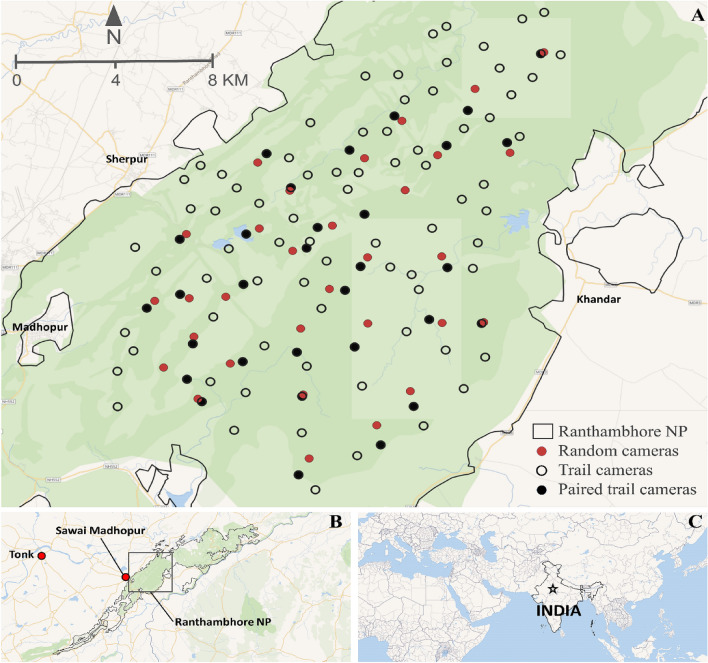
A. Locations of random and trail cameras placement within Ranthambhore National Park. The solid black circles represent trail cameras placed in the proximity of random cameras, i.e., paired trail cameras. *Inset: B. Study area extent in Ranthambhore Tiger Reserve (RTR); C. Location of RTR in India.* The maps were created using QGIS (*ver. 3.10,* https://download.qgis.org).

Photographs obtained from both the camera trap setup were archived and manually segregated to species. Since the number of trail cameras far exceeded the number of random cameras, we used only the estimates derived from paired cameras (one trail camera per site paired with a proximate random camera, distance range 90–900 m, Fig. 1) for meaningful and unbiased comparisons. Thus the richness and accumulation comparison was carried out using the data generated from 31 trail and 31 random cameras. Number of each species photo-captured by trail and random cameras were recorded to calculate species richness and accumulation. To compare species richness obtained from these two camera deployment designs, we generated sample-based species accumulation (richness) curve from incidence data^34^. Confidence intervals (95%) were computed based on unconditional variance following the method of Colwell et al.^35^, with 100 permutations. For both camera placement designs, species accumulation curves were computed based on the time taken to accumulate new species and reach an asymptote.

#### Relative abundance index

A careful scrutiny of each individual photo sequence was done to determine independent photo-capture events. Successive photo-captures (< 30 min apart) of the same species were considered as one event wherever the individual photo-captured animal(s) could not be identified with certainty (on the basis of gender, age class, and unique body markings). We used all random (n = 31) and trail (n = 106) cameras to calculate the Relative Abundance Index (RAI). In case of trail cameras, captures of the same individual at a location in both camera units were considered as a single capture (identified by the time of captures). The sampling effort was the sum of the number of days each camera was operational throughout the session; in case of trail setup, operational days of a camera station (camera station consists of two camera units facing each other on a trail) was considered for effort calculation. Species RAIs were calculated for trail and random cameras as the number of independent events of each species, divided by the total sampling effort of all the cameras multiplied by 100^5,36^ i.e. independent photo-capture events in 100 trap-nights.

Furthermore, we plotted robust density estimates of tigers and leopards obtained using spatially explicit mark recapture and ungulates obtained from line transect based distance sampling from Ranthambhore Tiger Reserve reported in^37^ against RAI values obtained from trail and random cameras. Since density estimates were cotemporaneous and from the same region, the scatter plot, scaling, and correlation between density estimates and RAI enabled us to evaluate the relationship between abundance and RAI and biases (if any) between different camera placements.

#### Detection probability

In order to estimate detection probability of species, we analyzed their presence /absence data within a multi-method occupancy framework^38^ where the two camera designs were taken as the two methods. For occupancy analysis, we used all the random (n = 31) and trail (n = 106) cameras as occupancy framework accounts for heterogeneous sampling effort while estimating the detection probability. Our aim was not to estimate the occupancy of the species in the study area, but to compare the detectability of the species by two camera trap placements. The sampling grids of 2 km^2^ were considered as the unit for occupancy analysis. The multi-method occupancy framework incorporates—(i) a local occupancy parameter (θ) (representing the probability of a region in the immediate vicinity of the camera is occupied), (ii) a site occupancy parameter (ψ) (describing the proportion of the sampling sites being occupied by the species during the study period), and (iii) detection probability (p^s^_t_, ‘s’ sampling method and ‘t’ occasion)^38^. Site-wise detection histories were made for each species using photo-captures obtained from the camera traps of both the sampling designs. Detection probabilities (occupancy estimation) were computed using the software PRESENCE^39^.

#### Activity pattern

Camera traps provide a non-invasive way to observe and quantify animal activity at the population level in a relatively cost-effective manner^11^. We used the time stamp metadata obtained from random (n = 31) and trail (n = 106) cameras to compute the activity pattern of wild ungulates and their major predators in the study area using the ‘overlap’ package in R^40^. ‘Overlap’ fits a kernel density function which corresponds to the photo-capture rate of the species in a time interval. The area under the curve (derived from kernel density function) represents the proportion of time the species was active. Frequency of camera trap images of a species in time reflect the activity of the species^41^. We estimated the degree of overlap (Δ—Delta^4^) between the wild ungulate activity recorded from random and trail cameras. Due to very few captures of carnivores in random cameras, we computed their activity only from trail cameras.

#### Group size

We calculated the group size of wild ungulates from the camera trap photo-captures. We counted the number of individuals of a species in an image and all images from the consecutive camera trap photos within 10 min to record group size. Any animal getting photo-captured after an interval of 10 min from the last photo-capture of the same species was considered as a member of a different group. We differentiated different individuals of a group by their physical characteristics and body markings to the best of our ability. Finally, we compared the frequencies of different group sizes observed from random and trail camera setups.

## Results

### Species richness and accumulation

**Figure 2:**
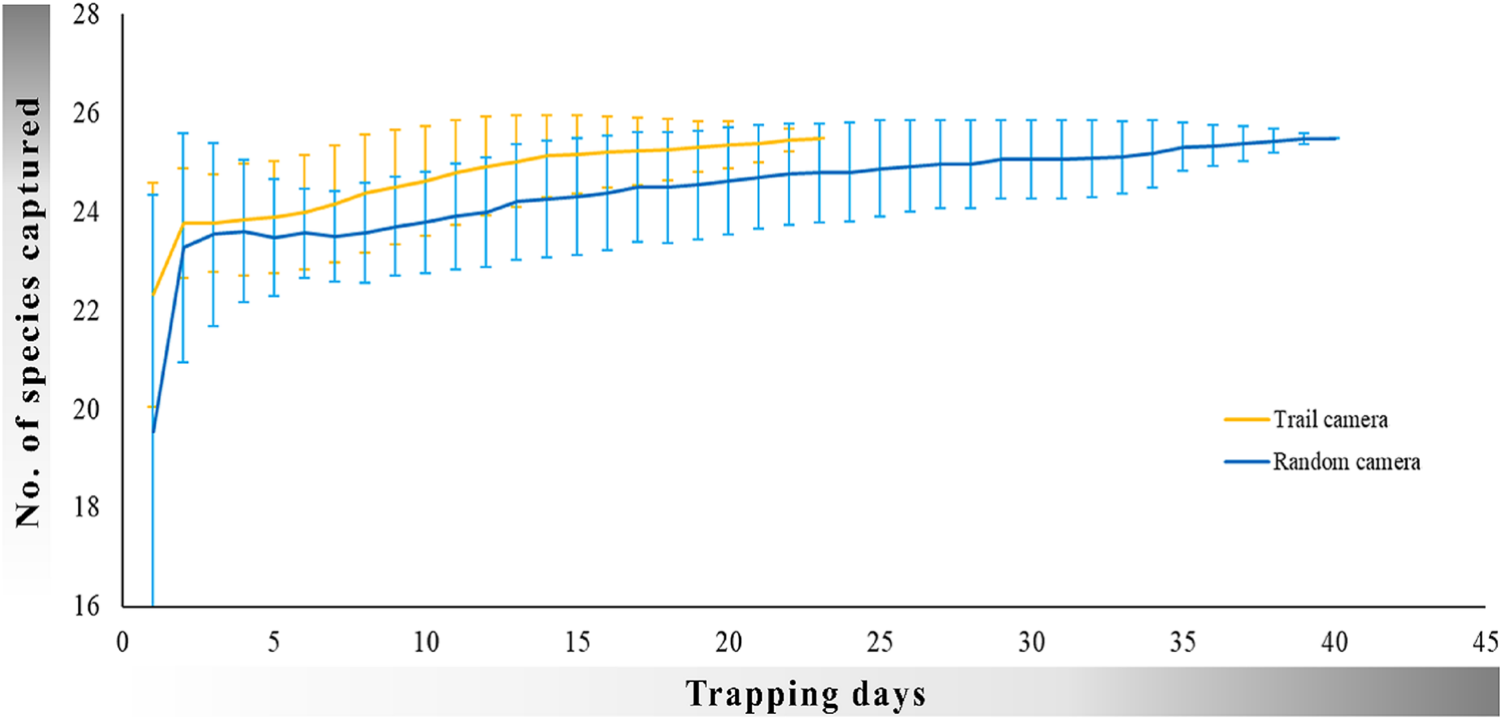
Species accumulation curves from trail (yellow line) and random (blue line) camera setups describing the rate at which species were captured in two setups. The vertical bars represent 95% confidence intervals.

A total number of 32 species were photo-captured in trail cameras (n = 106), and 25 species were photo-captured in random cameras (n = 31) (Table 1). However, both random and paired trail cameras (n = 31, trail cameras placed in the proximity of random cameras) detected 25 species (equal species richness), out of which 23 were common for both setups (Supplementary Table 1). The rate of species accumulation for trail camera setup was higher than that of random setup (Fig. 2).

**Table 1:** Number of species photo-captured, individual events, number of locations they were captured in (spatial capture), relative abundance index (RAI = 100 * Number of individual events/total effort), and detection probability (from occupancy analysis) obtained from trail (n = 106) and random (n = 31) camera setups.

Trophic level	Species	Trail camera setup					Random camera setup				
		No. of captures	No. of events	Spatial captures	RAI (± SE)	Detection probability (± SE)	No. of captures	No. of events	Spatial captures	RAI (± SE)	Detection probability (± SE)
Large carnivores	Tiger (*Panthera tigris*)	995	693	95	19.45 ± 1.66	0.194 ± 0.006	78	4	4	0.37 ± 0.18	0.003 ± 0.001
	Leopard (*Panthera pardus*)	180	131	59	3.69 ± 0.56	0.05 ± 0.005	81	10	5	1.02 ± 0.47	0.013 ± 0.004
	Striped hyaena (*Hyaena hyaena*)	528	456	80	13.1 ± 1.66	0.124 ± 0.006	158	18	10	1.75 ± 1.57	0.018 ± 0.004
	Sloth bear (*Melursus ursinus*)	162	97	54	2.8 ± 0.37	0.034 ± 0.004	46	7	6	0.46 ± 0.19	0.007 ± 0.003
Small carnivores	Golden jackal (*Canis aureus*)	57	36	12	1.2 ± 0.45	0.066 ± 0.011	9	2	2	0.17 ± 0.12	NA
	Indian fox (*Vulpes bengalensis*)	1	1	1	0.02	NA	–	–	–	–	–
	Jungle cat (*Felis chaus*)	235	213	58	5.96 ± 0.99	0.098 ± 0.005	55	5	3	0.35 ± 0.19	NA
	Desert cat (*Felis silvestris*)	1	1	1	0.02	NA	–	–	–	–	–
	Rusty-spotted cat (*Prionailurus rubiginosus*)	2	2	1	0.06	NA	–	–	–	–	–
	Honey badger (*Mellivora capensis*)	167	135	49	3.77 ± 0.67	0.073 ± 0.006	15	2	2	0.16 ± 0.11	NA
	Palm civet (*Paradoxurus hermaphrodites*)	116	100	35	2.88 ± 0.56	0.066 ± 0.005	17	4	2	0.29 ± 0.21	NA
	Small Indian civet (*Viverricula indica*)	53	46	25	1.24 ± 0.27	0.035 ± 0.005	8	2	2	0.26 ± 0.19	NA
	Ruddy mongoose (*Herpestes smithii*)	136	102	33	2.95 ± 0.64	0.043 ± 0.006	10	1	1	0.06 ± 0.06	NA
	Grey Mongoose *(Herpestes edwardsii*)	13	10	8	0.31 ± 0.11	NA	–	–	–	–	–
Herbivores	Spotted deer *(Axis axis*)	9825	1297	90	36.11 ± 4.65	0.28 ± 0.007	24,817	497	27	47.05 ± 14.6	0.277 ± 0.013
	Sambar (*Rusa unicolor*)	3068	921	94	25.76 ± 2.44	0.246 ± 0.007	15,796	380	30	39.75 ± 7.20	0.27 ± 0.012
	Blue bull (*Boselaphus tragocamelus*)	1047	445	60	13.63 ± 2.54	0.131 ± 0.006	1558	71	11	6.43 ± 2.83	0.083 ± 0.01
	Indian gazelle (*Gazella bennetti*)	101	48	9	1.19 ± 0.47	0.066 ± 0.009	139	12	2	0.85 ± 0.66	0.0675 ± 0.024
	Wild pig (*Sus scrofa*)	611	220	75	6.22 ± 0.77	0.074 ± 0.004	711	41	13	4.11 ± 1.51	0.0477 ± 0.007
Others	Porcupine (*Hystrix indica*)	697	567	86	15.73 ± 1.69	0.182 ± 0.006	432	29	11	2.72 ± 0.87	0.028 ± 0.005
	Hare (*Lepus nigricollis*)	1342	947	78	26.77 ± 3.71	0.283 ± 0.007	383	36	15	3.33 ± 0.81	0.042 ± 0.006
	Peafowl (*Pavo cristatus*)	4482	1939	97	53.04 ± 7.85	0.394 ± 0.008	1263	91	18	9.31 ± 2.12	0.075 ± 0.008
	Grey langur (*Presbytis entellus*)	1049	203	49	5.7 ± 1.00	0.822 ± 0.006	365	18	7	1.66 ± 0.76	0.022 ± 0.005
	Ground-dwelling birds (francolins, quails, etc.)	66	43	23	1.06 ± 0.35	NA	24	8	3	0.52 ± 0.32	NA
	Other birds (fliers)	55	46	27	1.34 ± 0.29	NA	2	2	1	0.23 ± 0.23	NA
	Fresh water crocodile (*Crocodylus palustris*)	1	1	1	0.02	NA	–	–	–	–	–
	Monitor lizard (*Varanus bengalensis*)	32	28	9	0.76 ± 0.27	NA	–	–	–	–	–
	Palm squirrel (*Funambulus palmarum*)	3	3	3	0.08 ± 0.05	NA	6	2	2	0.25 ± 0.14	NA
Domestic	Cattle (*Bos* spp.)	371	177	12	5.17 ± 3.13	0.055 ± 0.011	10	1	1	0.12	NA
	Camel (*Camelus dromedaries*)	4	4	3	0.10 ± 0.06	NA	–	–	–	–	–
	Donkey (*Equus* spp.)	1	1	1	0.02	NA	–	–	–	–	–
	Goat (*Capra* spp.)	–	–	–	–	–	26	2	1	0.13	NA
	Dog (*Canis lupus familiaris*)	3	2	2	0.05 ± 0.03	NA	1	1	1	0.06	NA

*SE* standard error, *NA* estimates not available.

### Relative abundance index

**Figure 3:**
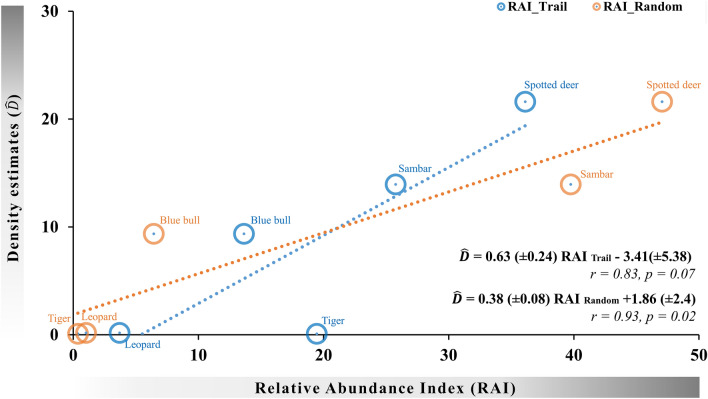
Scaling RAI values from different camera trap designs with absolute density. Only RAI’s from random camera trap placement designs had significant correlations with absolute density.

A total of 25,394 and 46,010 animal images were obtained from trail and random cameras, respectively. The relative abundance index (RAI) values of species obtained from random cameras were ordinated in the same order as absolute densities of these species while RAI of tigers and leopards from trail cameras were much higher than that from random cameras (Table 1, Fig. 3).

The RAI values obtained from random camera traps were highly correlated (r = 0.93, p < 0.05) with the density estimates obtained from SECR and distance sampling, while the RAI values from trail cameras were not significantly correlated (r = 0.38, p > 0.05) (Fig. 3) since tiger RAI was much higher. The linear equation depicting relationship between density and RAI obtained from random camera was:

$$ Density \, = \, 0.38 \, \left( { \pm 0.08} \right) \, RAI \, Random \, + 1.86 \, \left( { \pm 2.40} \right) $$

Here, a unit increase in density causes a 0.38-unit increase in RAI. The high correlation suggested that the relative abundance index obtained from random cameras can be used as a surrogate of abundance and also as an index to monitor trends in wildlife populations.

### Detection probability

Carnivore detection probability, obtained from occupancy estimation, was an order of magnitude higher on trail cameras (Table 1). It is noteworthy that herbivores detection probabilities in trail and random cameras were similar, whereas hare, porcupine, peafowl, and grey langur had higher detection probabilities on trail cameras (Table 1).

### Activity pattern

**Figure 4:**
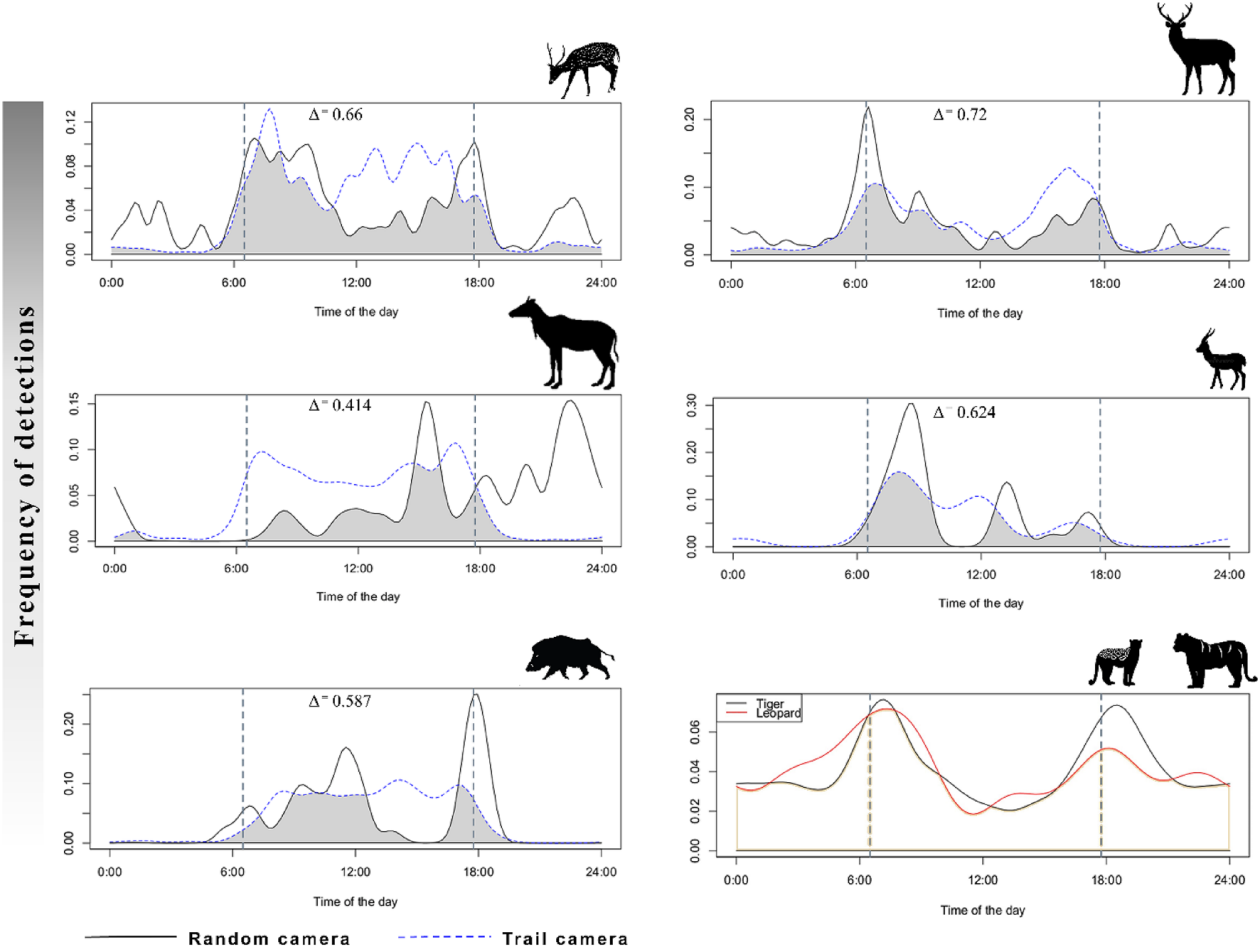
Activity pattern of wild ungulates (*L to R from top: spotted deer, sambar, blue bull, Indian gazelle, and wild pig*) and their major predators (*tiger and leopards*) in the study area. In each graph, the solid-black and dotted-blue line represents the species’ activity pattern obtained from random and trail cameras, respectively; the grey shaded polygons depicted the overlap between two curves. The vertical dotted gray line shows the timing of sunrise and sunset in the study area. Activity pattern of tigers and leopard was computed only from trail cameras.

According to the trail camera photo-captures, spotted deer, blue bull, wild pig, and Indian gazelle showed predominantly diurnal activity with very few captures at night, while sambar showed activity peaks in the early morning and late afternoon hours (Fig. 4). Contrastingly, data from random cameras showed spotted deer to have major activity peaks in the morning, with considerable photo-captures in the evening as well as at night. Sambar showed crepuscular activity peaks with night time activity in random cameras. The activity peaks for blue bull changed considerably in random cameras, where the species showed night time peak in activity (Fig. 3). Tiger and leopard showed nocturnal activity with crepuscular peaks from trail cameras; due to very less number of independent photo-captures in the random setup, we could not compute the temporal activity from random cameras for these carnivores. The trail cameras detected a higher percentage of activities for all the ungulates, except spotted deer, than random cameras (Table 2). Overlap values of Δ between random and trail cameras were least for blue bull (0.41 maximum difference) and highest for sambar (0.72 highest similarity) (Fig. 4) suggestive of substantial differences in estimates of percent time active between the two camera setups.

**Table 2:** Comparison of percent amount of time a species is active and estimates of group size of wild ungulates obtained from random and trail camera setups.

Species	Activity%		Group size					
	Random setup	Trail setup	Random setup			Trail setup		
	Mean (± SE)	Mean (± SE)	Sample size	Mean (± SE)	Range	Sample size	Mean (± SE)	Range
Spotted deer	39.50 (± 0.44)	31.53 (± 0.85)	582	2.63 (± 0.11)	1–20	1469	3.62 (± 0.11)	1–43
Sambar	19.04 (± 0.31)	32.28 (± 0.76)	416	1.76 (± 0.05)	1–9	968	1.66 (± 0.03)	1–12
Blue bull	27.03 (± 1.38)	38.76 (± 1.95)	70	1.60 (± 0.12)	1–5	361	1.49 (± 0.04)	1–8
Wild pig	16.47 (± 1.01)	39.18 (± 2.71)	43	1.44 (± 0.22)	1–4	227	1.70 (± 0.38)	1–8
Indian gazelle	13.63 (± 1.59)	26.16 (± 4.00)	14	1.85 (± 0.31)	1–5	49	1.26 (± 0.10)	1–5

### Group size

**Figure 5:**
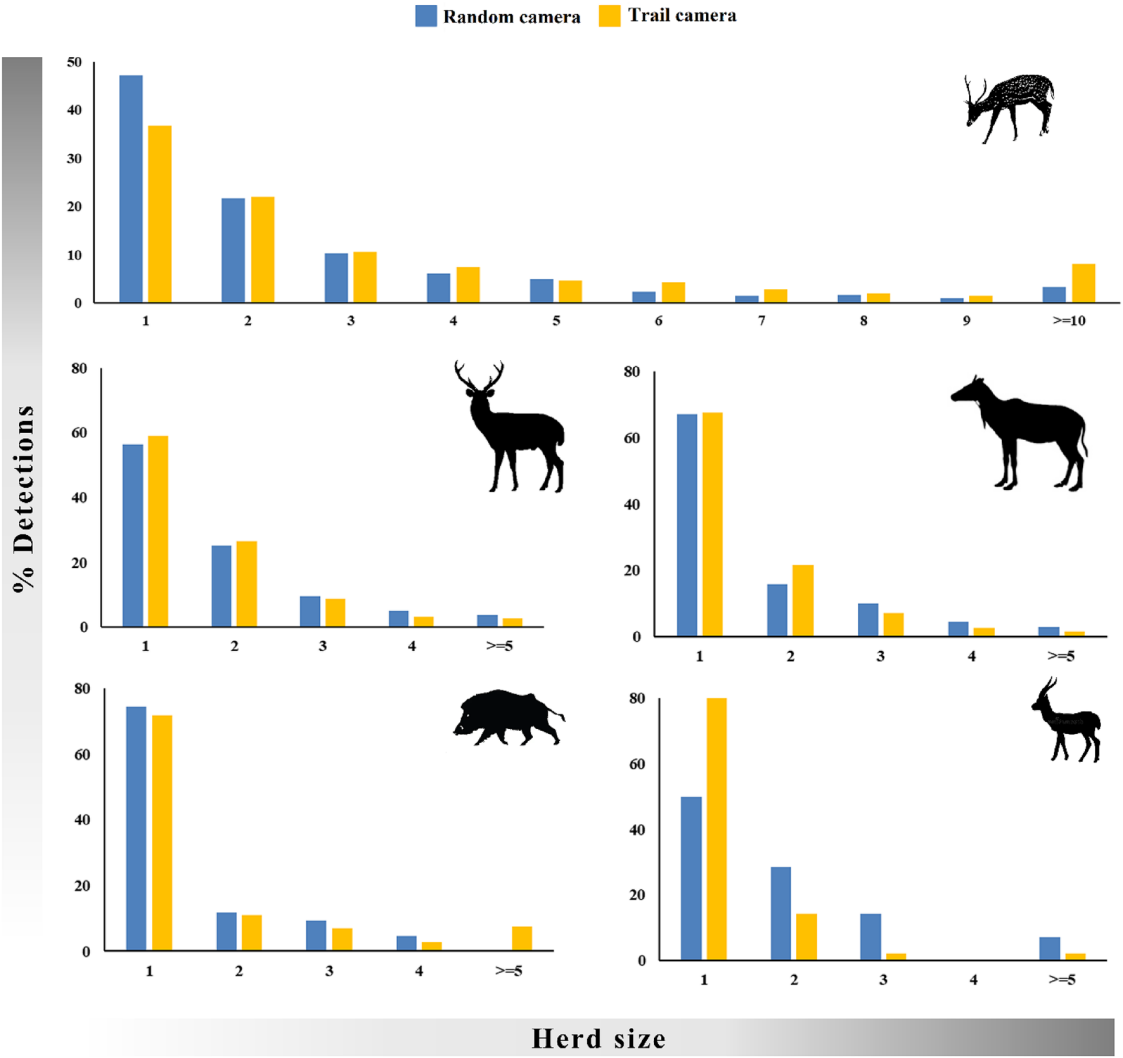
Herd size of wild ungulates (L to R from top: spotted deer, sambar, blue bull, wild pig, and Indian gazelle) recorded from the random and trail camera setups.

The average group sizes of all the ungulates obtained from both the camera setups were comparable, however, larger congregations were observed from trail cameras (Table 2). The frequency of different group sizes observed in random and trail cameras were comparable for all wild ungulate species (Fig. 5). Groups consisting of larger number of individuals were common in spotted deer, however, for sambar, blue bull, Indian gazelle, and wild pig single individuals were captured most frequently (Fig. 5).

## Discussion

Our results have implications on inferences of past studies and insights for planning future studies that use camera trap data to infer community composition, abundance, behavior and demographic parameter. Species accumulation curves act as a baseline to improve the efficiency of future community surveys^42^, therefore it is important to inquire about the optimal sampling design for this purpose. Species assemblages recorded in random and paired camera setups were the same, however, the rate of species accumulation was faster in trail camera setup than the random camera trap setup (Fig. 2). The above findings suggest that trail cameras placed for targeting large carnivore abundance estimation (in mark-recapture framework) can be used to generate species inventories in a short amount of time^28^.

Large carnivores, which occur at low density, patrol their territory using certain routes, were poorly captured in randomly placed camera traps (Table 1). Thus, the abundance indices of large carnivores obtained from random cameras were lower than that of the trail cameras. Smaller carnivores, like their larger counterparts, were significantly less represented in the randomly placed cameras. Moreover, it seems reasonable that carnivores (with soft pads) prefer mud roads or animal trails over random walk in a landscape with sharp pebbles and thorny vegetation. These findings were further endorsed by higher detection probability (Table1) (derived from multi-method occupancy analysis) of carnivores in trail cameras over the random cameras. Similar findings were published from the studies which found more carnivore captures on the trail compared to the off-trail and random cameras^26,28^. However, areas where man-managed road/trail densities are significantly low, studies did not find any differences in captures between trail and non-trail cameras^27^. In our opinion, if the objective is to assess relative abundance of various species within an ecosystem and compare these with density, then trail based RAI results are biased for large carnivores and random placement design results provide unbiased estimates of relative density (Fig. 3). However, if the objective of the study is to compare the relative abundance of the same species over time the use of trail-based camera placement would likely provide more precise estimates due to higher capture probability and therefore be more useful in detecting population trends. Caution should be exercised while comparing population trends using RAI values obtained from trail cameras, as detection rates can be influenced by camera placement, field expertise in choosing locations to maximize photo-captures and animal movement rates at these non-random selected locations^43^. Thus, bias may not remain consistent over different sampling intervals when using RAI obtained from trail cameras.

Ungulate species did not show significant differences in photo-capture rates from trail and random cameras (Table 1). Wild ungulates spend a large proportion of time foraging, and use trails mostly while moving from one foraging patch to another^44^. In consonance with our hypothesis, this explains the greater number of wild ungulate photo-captures in random cameras compared to trail cameras. While the average group size captured in both trail and random cameras were comparable, trail cameras recorded larger groups for ungulates (Table 2). This was likely as the species are known to move in bigger herds while they split into smaller sub-groups for foraging to avoid competition^45,46^. Although detection probabilities of wild ungulates were similar from the trail and random cameras, their activity patterns were substantially different. Trail cameras captured exclusively diurnal activity for all wild ungulate species, while the random cameras showed a more realistic activity pattern with records of night-time activity for spotted deer, sambar, and blue bull (Fig. 3). Trails are extensively used by predators during night, avoiding the use of trails at night was likely an anti-predatory behavior by wild ungulates^47,48^. Published activity patterns of these species have been obtained from trail cameras that focused on population estimation of large carnivores^22,23^ and therefore are likely biased towards diurnal activity.

Our study shows that both trail and random placement of cameras provide similar inference on species richness and composition, but trail cameras had faster accumulation rates and were therefore more cost-effective. Additionally, information on illegal activities inside the PA obtained from trail cameras were more comprehensive than random cameras. The relative abundance index (RAI) from both camera designs was similar for wild ungulates but much lower for carnivores in random setup. Our results for RAI suggest that random camera placement design is unbiased for estimates of relative abundance of species within a community, but biased data from trail cameras could still be used for estimating trends in abundance over time for any species within the same geographical area of sampling. Contrary to our findings, a few studies have reported the superiority of random camera setup over the trail-cameras for detecting rare species^27,28^, however, this was not the case for the semi-arid system that we studied. Activity patterns of ungulates and proportion of time active significantly differed between random and trail cameras. We propose that random cameras provide a more realistic representation of wild ungulate activity while trail cameras are better suited for estimating activity of carnivores.

Finally, no single method can address all the aspects concerning multiple species ecology or behavior, therefore camera trap surveys need to be tailor-made to cater to specific objectives^49^. Trail-based camera trapping is an important conservation tool for monitoring abundance of large carnivores that is required for their effective conservation. We show that ancillary data generated from this effort can additionally provide information on species richness, species specific trends in abundance and activity patterns of carnivores while inferences on activity patterns of ungulates from trail cameras can be biased.

## Supplementary Information

Supplementary Table 1.
