# Author Correction: Camera trap placement for evaluating species richness, abundance, and activity

**DOI:** 10.1038/s41598-022-05223-w

**Published:** 2022-01-13

**Authors:** Kamakshi S. Tanwar, Ayan Sadhu, Yadvendradev V. Jhala

**Affiliations:** Wildlife Institute of India, Chandrabani, Dehradun, 248001 India

Correction to: *Scientific Reports* https://doi.org/10.1038/s41598-021-02459-w, published online 29 November 2021

The original version of this Article contained an error in Figure 3, where the P-value reported at the end of the figure is incorrect,

“P = 0.001” and “P = 0.45”

now reads:

“P = 0.07” and “P = 0.02”

The original Figure 3 and accompanying legend appear below.

**Figure 3:**
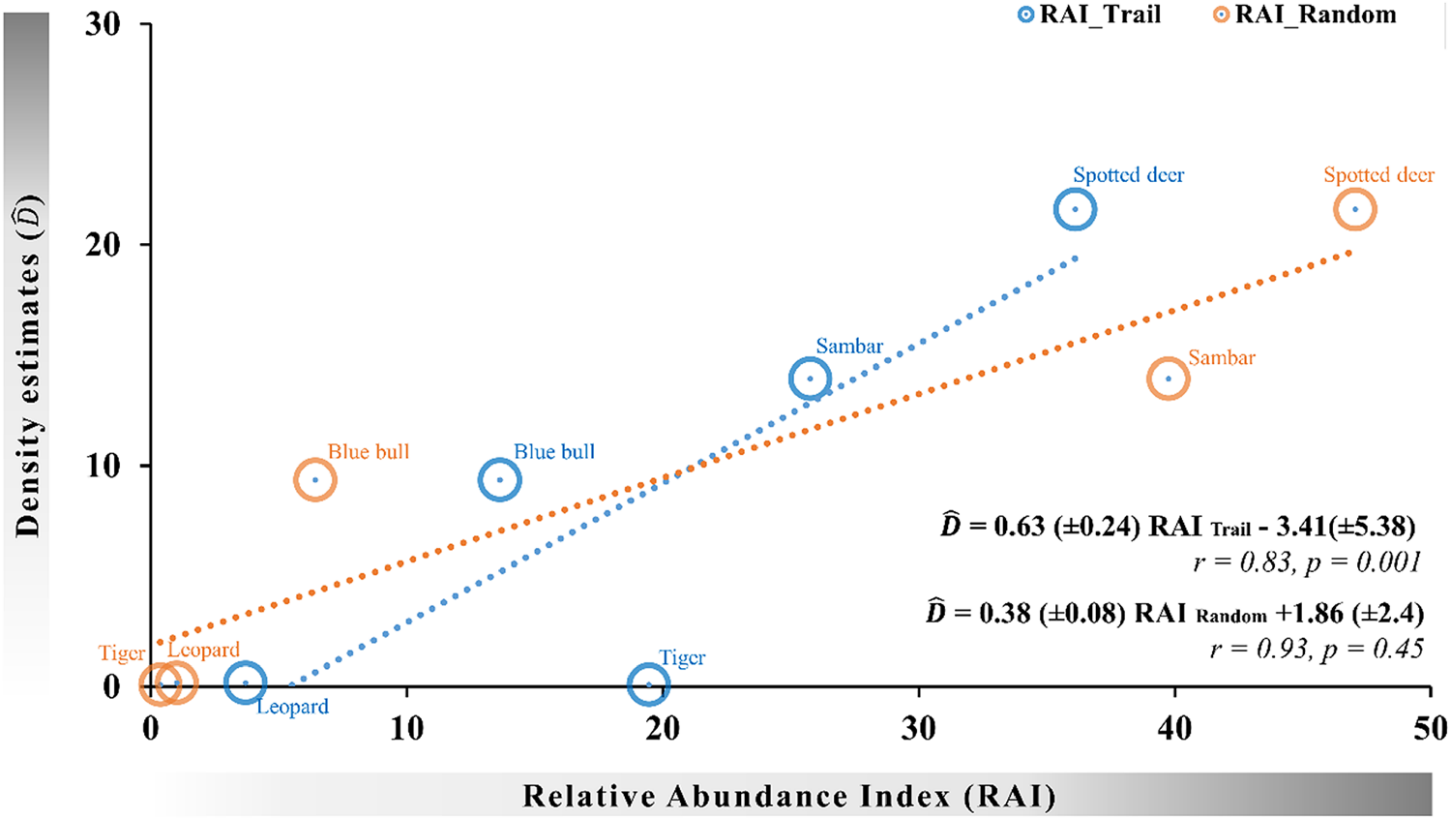
Scaling RAI values from different camera trap designs with absolute density. Only RAI’s from random camera trap placement designs had significant correlations with absolute density.

The original Article has been corrected.

