# Supplementary material for: Camera trap placement for evaluating species richness, abundance, and activity
Source: Sci Rep. 2021 Nov 29;11:23050. doi: 10.1038/s41598-021-02459-w (PMC8630032; doi:10.1038/s41598-021-02459-w)
Supplement: Supplementary file 1 — Supplementary Table 1. [file 41598_2021_2459_MOESM1_ESM.docx]

**Supplementary Table 1.** Number of species photo-captured in random cameras (n = 31) and paired trail cameras (n = 31) during the survey period in the study area. *Paired trail cameras =* *a trail camera paired with a proximate random camera*

| **S. No.** | **Species** | **Scientific Names** | **Random camera** | **Paired-trail camera** |
| --- | --- | --- | --- | --- |
| **1** | Tiger | *Panthera tigris* | *Yes* | *Yes* |
| **2** | Leopard | *Panthera pardus* | *Yes* | *Yes* |
| **3** | Striped hyaena | *Hyaena hyaena* | *Yes* | *Yes* |
| **4** | Sloth bear | *Melursus ursinus* | *Yes* | *Yes* |
| **5** | Golden jackal | *Canis aureus* | *Yes* | *Yes* |
| **6** | Jungle cat | *Felis chaus* | *Yes* | *Yes* |
| **7** | Palm civet | *Paradoxurus hermaphrodites* | *Yes* | *Yes* |
| **8** | Small Indian civet | *Viverricula indica* | *Yes* | *Yes* |
| **9** | Honey badger | *Mellivora capensis* | *Yes* | *Yes* |
| **10** | Ruddy mongoose | *Herpestes smithii* | *Yes* | *Yes* |
| **11** | Grey Mongoose | *Herpestes edwardsii* | *No* | *Yes* |
| **12** | Spotted deer | *Axis axis* | *Yes* | *Yes* |
| **13** | Nilgai | *Boselaphus tragocamelus* | *Yes* | *Yes* |
| **14** | Sambar | *Rusa unicolor* | *Yes* | *Yes* |
| **15** | Wild pig | *Sus scrofa* | *Yes* | *Yes* |
| **16** | Indian gazelle | *Gazella bennetti* | *Yes* | *No* |
| **17** | Porcupine | *Hystrix indica* | *Yes* | *Yes* |
| **18** | Hare | *Lepus nigricollis* | *Yes* | *Yes* |
| **19** | Peafowl | *Pavo cristatus* | *Yes* | *Yes* |
| **20** | Grey langur | *Presbytis entellus* | *Yes* | *Yes* |
| **21** | Ground-dwelling birds | francolins, jungle fowls, nightjars, partridges, quails, water hen, etc*.* | *Yes* | *Yes* |
| **22** | Other birds | babblers, crows, doves, drongos, kites, owls, robins, tree pie, etc. | *Yes* | *Yes* |
| **23** | Monitor lizard | *Varanus bengalensis* | *No* | *Yes* |
| **24** | Palm squirrel | *Funambulus palmarum* | *Yes* | *Yes* |
| **25** | Cattle | *Bos spp.* | *Yes* | *Yes* |
| **26** | Dog | *Canis lupus familiaris* | *Yes* | *Yes* |
| **27** | Goat | *Capra spp.* | *Yes* | *No* |
